# Cannabidiol Attenuates MK-801-Induced Cognitive Symptoms of Schizophrenia in the Passive Avoidance Test in Mice

**DOI:** 10.3390/molecules26195977

**Published:** 2021-10-02

**Authors:** Marta Kruk-Slomka, Grazyna Biala

**Affiliations:** Department of Pharmacology and Pharmacodynamics, Medical University of Lublin, 4a Chodzki Str., 20-093 Lublin, Poland; grazyna.biala@umlub.pl

**Keywords:** schizophrenia, cognitive disorders, memory and learning, cannabidiol, mice, passive avoidance test

## Abstract

Schizophrenia is a chronic mental disorder that disturbs feelings and behavior. The symptoms of schizophrenia fall into three categories: positive, negative, and cognitive. Cognitive symptoms are characterized by memory loss or attentional deficits, and are especially difficult to treat. Thus, there is intense research into the development of new treatments for schizophrenia-related responses. One of the possible strategies is connected with cannabidiol (CBD), a cannabinoid compound. This research focuses on the role of CBD in different stages of memory (acquisition, consolidation, retrieval) connected with fear conditioning in the passive avoidance (PA) learning task in mice, as well as in the memory impairment typical of cognitive symptoms of schizophrenia. Memory impairment was provoked by an acute injection of the N-methyl-D-aspartate (NMDA) receptor antagonist MK-801 (animal model of schizophrenia). Our results revealed that an acute injection of CBD (30 mg/kg; intraperitoneally (i.p.) improved all phases of long-term fear memory in the PA test in mice. Moreover, the acute injection of non-effective doses of CBD (1 or 5 mg/kg; i.p.) attenuated the memory impairment provoked by MK-801 (0.6 mg/kg; i.p.) in the consolidation and retrieval stages of fear memory, but not in the acquisition of memory. The present findings confirm that CBD has a positive influence on memory and learning processes in mice, and reveals that this cannabinoid compound is able to attenuate memory impairment connected with hypofunction of glutamate transmission in a murine model of schizophrenia.

## 1. Introduction

Schizophrenia is a chronic disease with a rising incidence in recent years. It is defined as a mental illness with an individual course and non-specific symptoms. The symptoms of schizophrenia fall into three categories: positive (hallucinations, visions), negative (withdrawal, decreased activity and increased demotivation), and cognitive (memory loss, concentration disorders) [1,2].

The treatment of schizophrenia is not easy, as its etiology is not yet fully understood. One of the hypotheses for the development of schizophrenia is the glutamate(Glu)-related hypothesis. Glu is the main excitatory neurotransmitter in the brain, and has a leading role in neural physiology. Because of its role in synaptic plasticity, Glu is involved in cognitive functions in the brain, such as learning and memory. Additionally, it has been revealed that glutamatergic transmission through N-methyl-D-aspartate (NMDA)-type receptors is strictly implicated in specific symptoms of schizophrenia, including cognitive-related problems [3,4].

In order for the therapy of schizophrenia to be effective, it requires a long-term use of specific drugs. The pharmacotherapy of schizophrenia is based on antipsychotics (neuroleptics), divided into two generations: the first generation includes older (classic) antipsychotics, while the second generation represents newer, atypical neuroleptics. The main difference between these types lies in their side effects, which are more frequent in the first generation antipsychotics. The observed side effects of antipsychotics may include the following: dyskinesia (uncontrollable movements of the jaw, the lips or the tongue), akathisia (uncomfortable restlessness), headaches, dizziness, diarrhea, anxiety, sedation, sexual problems, weight gain, constipation, dry mouth, hyperprolactinemia, blurred vision, etc.

It has also been noted that the treatment of schizophrenia is exclusively based on the control of symptoms; therefore, it is not possible to completely cure the patient with available antipsychotic drugs. The goal of the available pharmacotherapy is to achieve remission, i.e., a state in which the affected patient can live normally, without experiencing the unpleasant symptoms of the disease. However, the available pharmacological programs induce a good response to treatment in 40–50% of patients, while leaving 30–40% as partial and 10–30% as non-responders to available antipsychotics. The currently used neuroleptics enable a fairly effective control of positive symptoms, while their efficacy in alleviating negative and cognitive symptoms is largely limited and negligible, respectively [5].

Thus, intense research has been carried out with the aim of developing new treatments to improve therapy responses, especially in the case of cognitive symptoms. One of the possible strategies to modulate cognitive symptoms of schizophrenia is based on cannabinoids and the endocannabinoid system (ECS) [6,7,8].

The ECS, through its cannabinoid type 1 (CB1) and 2 (CB2) receptors, can modulate many physiological functions, including different aspects of memory-related processes, anxiety, pain, and psychosis. Thus, a modulation of the ECS’s functions through specific compounds (cannabinoids) with affinity for CB receptors could open new perspectives for CB-based therapies.

Cannabinoids are a group of compounds (more than 100) derived from the Cannabis sativa plant, and used for centuries to treat different conditions, such as anxiety, pain, depression, dementia, psychosis, or other schizophrenia-like symptoms. On the one hand, cannabis use has been associated with an increased risk of schizophrenia, as the main psychoactive phytocannabinoid (Δ9-tetrahydrocannabinol, Δ9-THC) induces dose-dependent psychiatric symptoms, such as psychosis or memory impairment [8,9]. On the other hand, however, cannabidiol (CBD) is another major non-psychotropic constituent of Cannabis sativa that has attracted growing attention in recent years. Currently, there are more than 100 registered clinical trials being carried out to confirm the therapeutic effects of CBD, including its potentially antipsychotic effects, or properties beneficial for the restoration of cognitive processes [10,11,12,13,14,15].

Despite a great amount of literature dealing with the influence of CBD on memory and learning processes [11,12,13], there are few available data describing the influence of CBD on the cognitive-related symptoms of schizophrenia; thus, the mechanism of this function remains unclear.

Hence, the aim of our research was to evaluate the beneficial effects of CBD for mice with cognitive disturbances, in the context of cognitive symptoms of schizophrenia. Following that, we attempted to investigate, for the first time, the influence of CBD on the three different stages (acquisition, consolidation, and retrieval) of long-term memory-related processes connected with fear conditioning in the passive avoidance (PA) learning task in mice. In addition, based on the most well-known hypothesis for the etiology of schizophrenia—i.e., the glutamate(Glu)-related hypothesis—the aim of the present study was to evaluate the influence of CBD on cognitive-related symptoms in mice, using a pharmacological animal model of schizophrenia. Memory impairment, typical of the cognitive symptoms of schizophrenia, was provoked by an acute injection of the N-methyl-D-aspartate (NMDA) receptor antagonist MK-801 (the animal model of schizophrenia) [16].

We hope that our findings may broaden the knowledge of the role of CBD in memory processes in the context of schizophrenia-like responses in mice, including the interactions between CBD and other receptors strictly associated with schizophrenia, e.g., NMDA receptors. Furthermore, the assessment of the role of CBD in this type of memory (fear memory in the PA test) seemed for the first time to also be important in the context of other symptoms of schizophrenia (e.g., anxiety), as well as of side effects after neuroleptics. Thus, our results may help to develop new, CBD-based pharmacological strategies—especially, but not only, to control the cognitive symptoms of schizophrenia in humans.

## 2. Results

### 2.1. Memory-Related Responses

First, we evaluated the influence of an acute administration of CBD on the different stages of long-term memory: acquisition, consolidation, and retrieval.

#### 2.1.1. The Influence of an Acute Injection of CBD on the Long-Term Memory in Mice in the PA Test

##### Acquisition of Memory

**Figure 1:**
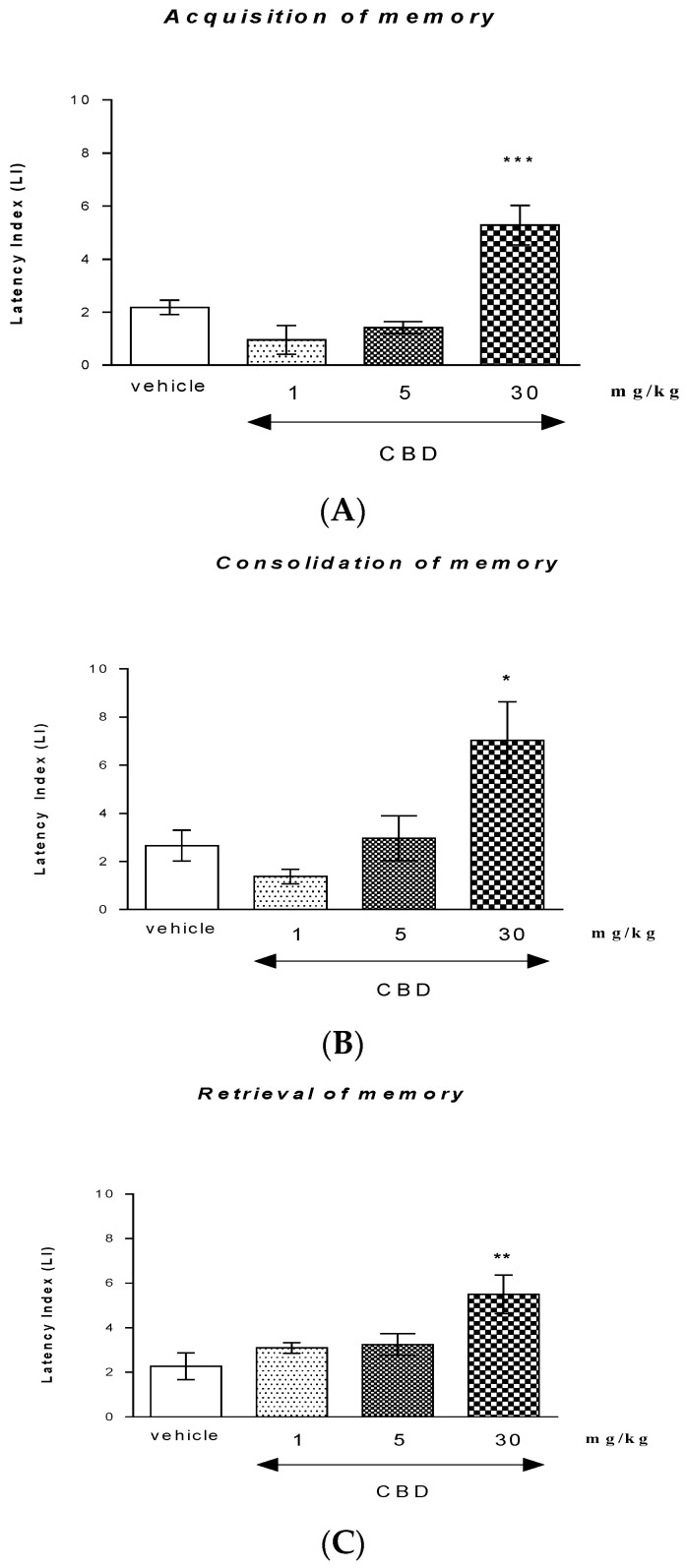
Influence of an acute administration of CBD on the cognition-related responses, expressed as latency index (LI), during the acquisition (**A**), consolidation (**B**), and retrieval (**C**) of memory using the PA test in mice. CBD (1, 5, or 30 mg/kg) or vehicle were administered 30 min before the first trial (acquisition of memory), immediately after the first trial (consolidation of memory), or 30 min before the second trial (retrieval of memory). The second trial was conducted 24 h after the first; *n* = 8–10; the means ± SEM; * *p* < 0.05; ** *p* < 0.01; *** *p* < 0.001 vs. vehicle-treated group; Tukey’s test.

One-way ANOVA revealed that the administration of acute i.p. doses of CBD (1, 5, and 30 mg/kg) had a statistically significant effect on LI values for long-term memory acquisition (F (3.33) = 15.99; *p* < 0.0001). Indeed, Tukey’s post hoc test confirmed that only CBD at the highest dose (30 mg/kg) significantly increased LI values in mice compared to those in the vehicle-treated control group (*p* < 0.001) (Figure 1A), indicating that CBD, at this used dose, improved the long-term acquisition of memory and learning processes in the PA test in mice.

##### Consolidation of Memory

One-way ANOVA revealed that the administration of acute i.p. doses of CBD (1, 5, and 30 mg/kg) had a statistically significant effect on LI values for long-term memory consolidation (F (3.31) = 6.105; *p* = 0.0025). Indeed, Tukey’s post hoc test confirmed that only CBD at the highest dose (30 mg/kg) significantly increased LI values in mice compared to those in the vehicle-treated control group (*p* < 0.05) (Figure 1B), indicating that CBD, at this used dose, improved the long-term consolidation of memory and learning processes in the PA test in mice.

##### Retrieval of Memory

One-way ANOVA revealed that the administration of acute i.p. doses of CBD (1, 5, and 30 mg/kg) had a statistically significant effect on LI values for long-term memory consolidation (F (3.31) = 5.473; *p* = 0.0043). Indeed, Tukey’s post hoc test confirmed that only CBD at the highest dose (30 mg/kg) significantly increased LI values in mice compared to those in the vehicle-treated control group (*p* < 0.01) (Figure 1C), indicating that CBD, at this used dose, improved the long-term retrieval of memory and learning processes in the PA test in mice.

In the next step, we assessed the impact of CBD on the memory impairment provoked by an acute injection of MK-801 during these three stages of long-term memory. Based on the results obtained from the above-described pilot experiments, the non-effective doses of CBD (1 and 5 mg/kg) were then chosen for the next behavioral experiments, evaluating the influence of this cannabinoid compound on the memory impairment provoked by an acute injection of MK-801 (0.6 mg/kg), using the PA test in mice.

#### 2.1.2. The Influence of the Administration of CBD on the Memory Impairment Provoked by an Acute Administration of MK-801 in the PA Test in Mice

##### Acquisition of Memory

**Figure 2:**
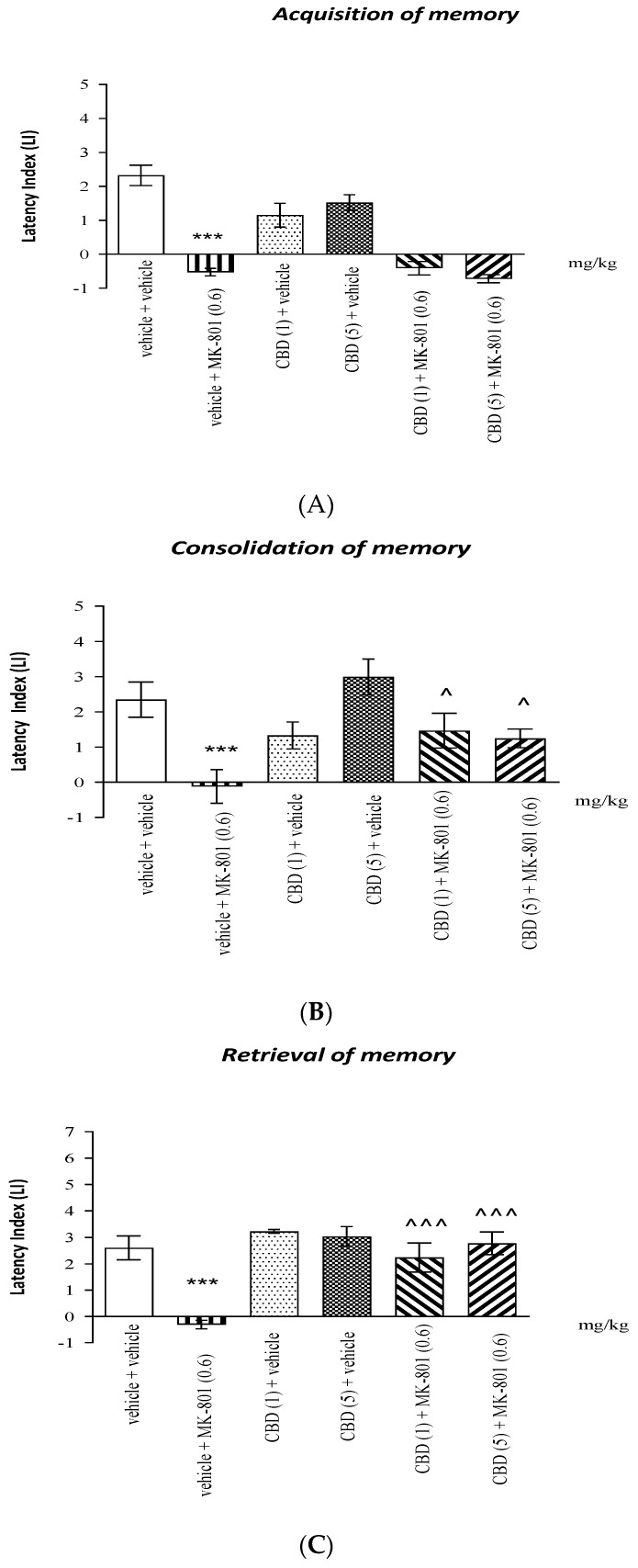
Influence of an acute co-administration of CBD (1 or 5 mg/kg) and an effective (amnestic) dose of MK-801 (0.6 mg/kg) on the cognition-related responses, expressed as latency index (LI), during the acquisition (**A**), consolidation (**B**), and retrieval (**C**) of memory using the PA test in mice. Non-effective doses of CBD (1 or 5 mg/kg) or saline were administered 15 min prior to the amnestic dose of MK-801 (0.6 mg/kg). Last injections were performed 15 min before the first trial (acquisition of memory), immediately after the first trial (consolidation of memory), or 15 min before the second trial (retrieval of memory). The second trial was conducted 24 h after the first; n = 8–9; the means ± SEM; *** *p* < 0.001 vs. vehicle group; ^ *p* < 0.05; ^^^ *p* < 0.001 vs. saline/MK-801 (0.6 mg/kg)-treated group; Tukey’s test.

For long-term memory acquisition, two-way ANOVA revealed that there was a statistically significant effect caused by MK-801 (0.6 mg/kg) treatment (F (1.42) = 134.8; *p* < 0.0001), as well as a statistically significant effect caused by interactions (F (2.42) = 3.784; *p* = 0.0308), but there was no statistically significant effect caused by CBD (1 or 5 mg/kg) pretreatment (F (2.42) = 3.207; *p* = 0.0505). Tukey’s post hoc test confirmed that MK-801 at the dose of 0.6 mg/kg significantly decreased LI values in mice in the PA test in comparison to the vehicle-treated mice, pointing to the amnestic effect of this drug (*p* < 0.001). Moreover, an acute injection of CBD (1 or 5 mg/kg) had no influence on the amnestic effect of MK-801 (0.6 mg/kg) (*p* > 0.05; Tukey’s test) (Figure 2A).

##### Consolidation of Memory

For long-term memory consolidation, two-way ANOVA revealed that there was a statistically significant effect caused by MK-801 (0.6 mg/kg) treatment (F (1.44) = 13.77; *p* = 0.0006), as well as a statistically significant effect caused by interactions (F (2.44) = 4.643; *p* = 0.0148), but there was no statistically significant effect caused by CBD (1 or 5 mg/kg) pretreatment (F (2.44) = 2.571; *p* = 0.0879). Tukey’s post hoc test confirmed that MK-801 at the dose of 0.6 mg/kg significantly decreased LI values in mice in the PA test in comparison to the vehicle-treated mice, pointing to the amnestic effect of this drug (*p* < 0.001). Moreover, an acute injection of both doses of CBD (1 or 5 mg/kg) attenuated the amnestic effect of MK-801 (0.6 mg/kg) (*p* < 0.05; Tukey’s test) (Figure 2B).

##### Retrieval of Memory

For long-term memory retrieval, two-way ANOVA analyses revealed that there was a statistically significant effect caused by MK-801 (0.6 mg/kg) treatment (F (1.42) = 20.27; *p* < 0,0001), as well as a statistically significant effect caused by interactions (F (2.42) = 6.581; *p* = 0.033) and a statistically significant effect caused by CBD (1 or 5 mg/kg) pretreatment (F (2.42) = 13.19; *p* < 0.0001). Tukey’s post hoc test confirmed that MK-801 at the dose of 0.6 mg/kg significantly decreased LI values in mice in the PA test in comparison to the vehicle-treated mice, pointing to the amnestic effect of this drug (*p* < 0.001). Moreover, an acute injection of both doses of CBD (1 or 5 mg/kg) significantly attenuated the amnestic effect of MK-801 (0.6 mg/kg) (*p* < 0.001; Tukey’s test) (Figure 2C).

### 2.2. Locomotor Activity

**Figure 3:**
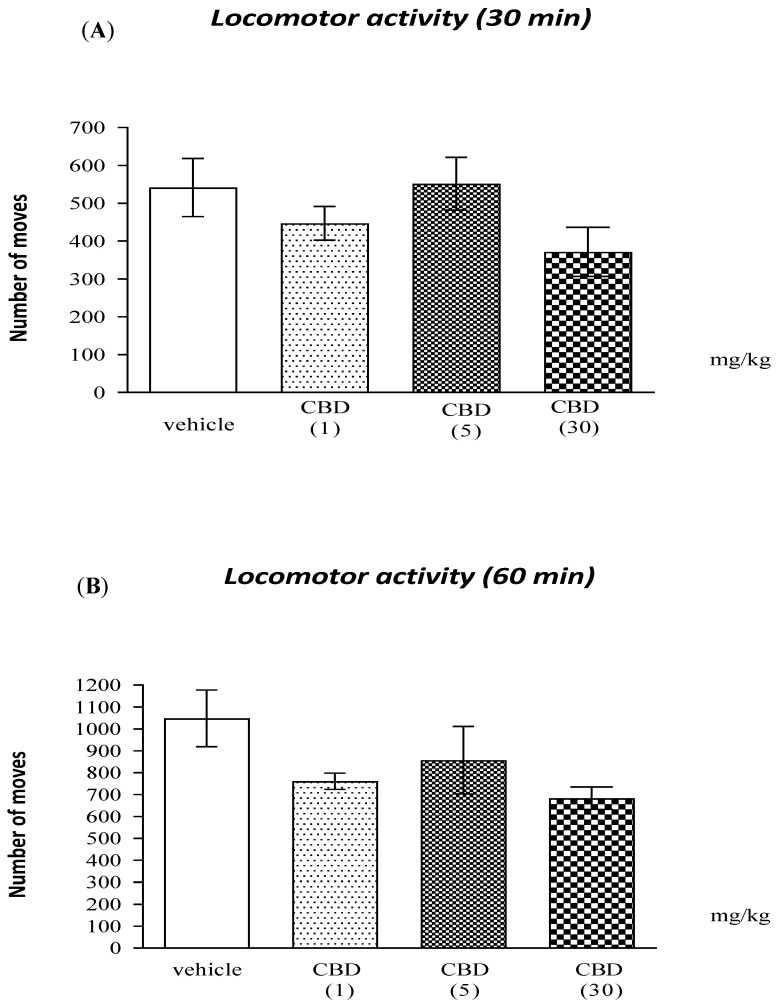
Effects of CBD on locomotor activity. The data are shown as the means ± SEM; photocell beam breaks of mice measured immediately after injection for 30 min (**A**) and 60 min (**B**); *n* = 8–10.

One-way ANOVA revealed that CBD (1, 5, 30 mg/kg) at the doses tested, caused no statistically significant changes in the locomotor activity assessed for 30 minutes of the experiment (F (3.31) = 1.704; *p* = 0.1888) or after 60 min: (F (3.31) = 2.228; *p* = 0.1070) (Figure 3A,B, respectively).

## 3. Discussion

The correlation between ECS and memory-related effects has long been a matter of debate. Several lines of preclinical and clinical evidence point to a very strong relationship between the modulation of ECS function and memory and learning processes [1]. It has been reported that CB1 receptor agonists provoke memory-related disturbances [17,18], whereas the antagonists of this type of receptor improved memory and learning processes in rodents evaluated in many memory tasks [17,19,20]. However, the role of the CB receptor ligand in the cognitive symptoms of schizophrenia has not yet been fully elucidated, and is thus still unclear.

Of the CB receptor ligands, CBD—a natural, bioactive compound of Cannabis sativa with a complex mechanism of action—was selected to be the target of our research. CBD shows low affinity for CB1 and CB2 receptors, and is capable of altering the functions of CB receptors by antagonizing the effects of CB1 and CB2 receptor agonists [19,21]. Likely due to its mechanism of action, CBD reveals a promising therapeutic potential for a broad range of neurological and psychiatric conditions, including memory disturbances [22].

Thus, based on the above-mentioned data from the literature, the purpose of the experiments presented herein was to explore the role of CBD in various contexts of cognition.

We first examined the influence of CBD on the different stages of long-term (acquisition, consolidation, and retrieval) memory-related responses and, in a subsequent step, we tried to determine CBD’s involvement in MK-801-induced memory impairment in mice, which corresponds to the cognitive symptoms of schizophrenia in humans. In order to assess cognitive function after CBD and CBD/MK-801 injections, we applied the passive avoidance (PA) test, commonly used in pharmacological studies.

### 3.1. CBD Impacts on Different Memory Stages

In the first step, we tried to test the effects of various CBD doses (1–30 mg/kg) on different memory stages in the PA test because, as the literature data suggest, many effects of CBD—such as memory-related or -unrelated process effects—are dose-dependent and associated with the injection–test time interval, the route of administration, and the applied behavioral test. Our studies revealed that only an acute injection of CBD at its highest dose (30 mg/kg) was able to significantly improve long-term fear memory acquisition, consolidation, and retrieval in the PA test in mice, while CBD doses of 1 and 5 mg/kg were ineffective. Moreover, in order to confirm or exclude the influence of locomotion on the data obtained in our memory-related behavioral experiments, we attempted to evaluate the impact of an acute administration of CBD on horizontal locomotion in mice. Our results showed that an acute administration of CBD (at any dose) had no influence on the locomotor activity of the mice.

In our behavioral experiments, we used the PA task—a procedure that allows for the examination of long-term associative memory at different stages (acquisition, consolidation, and retrieval)—with regard to the time of drug treatment. Our findings remain in line with other literature data concerning the modulatory actions of CBD on different memory types, but no available studies to date have described the influence of CBD on all of the memory phases.

In general, literature data suggest that CBD improves memory and learning processes. There is preclinical and clinical evidence for the potential of CBD to regulate different memory types. For example, chronic use of CBD has been shown to improve working memory in the T-maze test, and to increase the preference for a novel object in the novel object recognition (NOR) test. [23,24,25,26]. Research has also shown that acute administration of CBD affects the so-called fear memory [11,24,25,26]. One such experience describes how a systemic acute administration of CBD before fear conditioning resulted in attenuated fear expression during later memory retrieval testing [9], indicating that the acute administration of CBD compromised the acquisition of fear learning. Similarly, in our experiments, the acute administration of CBD improved the acquisition and consolidation of fear memory in the PA test. In contrast, there have only been a few reported effects of chronic CBD injections on fear memory expression, and the available reports on the issue tend to conflict with one another. Chronic daily injections of CBD (for 14 days), administered prior to conditioning, enhanced fear expression during retrieval testing, thus showing memory improvement and, consequently, suggesting that chronic CBD facilitated fear learning [27]. In contrast, another study showed no such effect of CBD chronic administration (for 21 days) on fear conditioning, suggesting no CBD potential for this type of memory [28]. Moreover, contrary to the reported positive effects of CBD on fear extinction, a systemic administration of CBD has been shown to impair the reconsolidation of contextual fear memory after its brief retrieval [29,30]. This CBD-induced negative effect on reconsolidation required its administration immediately after memory retrieval. Interestingly, a systemic injection of CBD had no effect if it was given without—or within 6 h after—retrieval. Additionally, CBD was also able to stop the reconsolidation of both older and newer fear memories [29].

There are also studies that provide direct evidence to support the ability of CBD to attenuate some of the cognitive-impairing effects of Δ9-THC (the primary psychoactive constituent of cannabis). Wright et al. revealed that CBD has the potential to attenuate the negative effects on associative memory provoked by Δ9-THC [31].

Although the supporting effect of CBD on memory processes has already been demonstrated, there are still no available data regarding the influence of CBD on the cognitive-related symptoms of schizophrenia. Since the effects of CBD on memory and learning were dose-dependent in our study, and brought about memory improvement, it would be interesting to investigate the effects of CBD on memory impairment, typical of schizophrenia, in order to unveil and evaluate CBD’s mechanism of action and to reveal, in the context of our experiments, the potential effectiveness of CBD against dysfunction of NMDA receptors.

In the second step, based on the results obtained from the first experiments, we proceeded to check the influence of CBD on the MK-801-provoked cognitive disturbances connected with fear conditioning in the PA learning task in mice. Inactive doses of CBD (1 and 5 mg/kg) were chosen for the experiment dealing with the administration of MK-801 (0.6 mg/kg), in order to show the possible antagonistic effects of CBD on the amnestic effects of MK-801.

### 3.2. CBD’s Effects on MK-801-Induced Memory Impairment

We attempted to assess, for the first time, the influence of acute CBD injections on memory impairment provoked by MK-801 in mice. We found that an acute injection of CBD at non-effective doses (1 and/or 5 mg/kg) attenuated the consolidation and restoration of long-term fear memory impairment, while an acute administration of CBD had no influence on MK-801 memory impairment during the acquisition phase in the PA test in mice.

It should be emphasized that the earlier measurement of CBD’s effects on locomotor activity, as well as the use of ineffective CBD doses in the PA tests, prevent us from concluding that the reversal of MK-801-induced memory disorders in mice (in the PA test) by CBD (1 and 5 mg/kg) was either a false positive or a false negative result.

The examinations of the preclinical efficacy of CBD in treating/controlling cognitive disturbances in neuropsychiatric disorders—e.g., during schizophrenia—are rather limited [22]. Consequently, there are few available data describing the influence of CBD on the cognitive-related symptoms of schizophrenia. One such relevant study is an experiment in which the researchers used rats prenatally infected with the poly I:C virus as a model of schizophrenia. The administration of poly I:C during mid-to-late pregnancy induced less sociability and other (negative and cognitive) symptoms of schizophrenia in the offspring. The administration of CBD in the offspring improved social interactions in the group [32].

Other studies described the role of CBD in modulating other types of memory, such as novel object and social recognition, in cognitively impaired animals [28,33]. Fagherazzi et al. [33] used an animal model of cognitive impairment induced by iron overload. Both an acute injection of CBD at the highest dose and a chronic administration of CBD improved recognition memory in iron-treated rats in the NOR task. Neither acute nor chronic CBD doses affected memory in control rats [33].

Our results are consistent with other available preclinical studies describing the positive influence of CBD on various disturbances provoked by the preclinical schizophrenia model of NMDA receptor hypofunction. A systemic administration of CBD was able to attenuate MK-801-induced hyperactivity, deficits in pre-pulse inhibition (PPI), and social withdrawal [34]. In addition, an injection of CBD attenuated MK-801-induced prolonged PPI in a murine sensory gating model, similarly to clozapine—a drug used in the treatment of schizophrenia [35]. In another study with MK-801 (1 mg/kg), mice were administered CBD for 22 days and then examined in the NOR test. The discrimination index, measured in the NOR test, was significantly higher in the MK-801-treated mice administered with the highest dose of CBD (60 mg/kg), compared to the control group [36]. Contrary to the results cited above, Deiana et al. [37] found that an acute injection of CBD prior to MK-801 (0.08 mg/kg) did not prevent the MK-801-provoked deficits in social recognition memory, while administration of CBD to control rats had had no significant effect on social recognition [37]. The results from the cited studies indicate that high doses of CBD may alleviate the dysfunction of object recognition memory, but not social recognition memory, as observed in the MK-801-related rodent model of schizophrenia.

As we have mentioned before, CBD, along with Δ9-THC, is the most abundant bioactive compound of Cannabis sativa. Δ9-THC demonstrates strong psychotic properties, while, in contrast to Δ9-THC, CBD is devoid of any psychotropic effects [38].

The psychotropic effects of Δ9-THC are due to the activation of the CB1 receptor, which is one of the receptors most highly expressed in the central nervous system, where it mediates the signaling of the endocannabinoids in the brain: N-arachidonoylethanolamine (AEA) and 2-arachidonoylglycerol (2-AG). Moreover, the activation of the CB1 receptor acts as negative regulation of the NMDA receptor, causing its hypofunction [39,40]. The dysfunction of the NMDA receptor is associated with the dopaminergic dysregulation observed in schizophrenic patients, giving rise to the hypothesis that glutamatergic/NMDA dysfunction underlies schizophrenic symptoms, including psychosis and memory impairment.

CBD’s effects on memory and learning are dose-dependent, and are connected with the ECS system. CBD shows a low affinity for the CB1 and CB2 receptors, and is capable of altering the functions of CB receptors by antagonizing CB1 and CB2 receptor agonists, such as AEA and 2-AG [21,41]. CBD acts as a negative allosteric modulator on the CB1 receptor [41], which could explain—at least in part—its balancing effects on the psychotic effects of Δ9-THC. Moreover, CBD is able to interfere with the effects of Δ9-THC, suggesting that the Δ9-THC/CBD ratio in cannabis might moderate the adverse effects after its consumption. Additionally, Leweke et al. [42] and Bisogno et al. [43] found that CBD increased the levels of AEA, reducing cellular uptake and inhibiting hydrolytic degradation of AEA [42,43]; thus, they presented a hypothesis describing the role of AEA in counteracting the overactivity of dopamine D2 receptors. For this reason, we may assume that CBD could have antipsychotic properties, enhancing the synaptic AEA level to rebalance the D2 receptor overactivation. In addition, CBD suppresses the expression and activity of fatty acid amide hydrolase (FAAH)—an enzyme required for the degradation of both AEA and 2-AG [14,15,44].

However, the underlying mechanism of the involvement of CBD in the MK-801-induced memory impairment presented in our experiments remains unclear. It is possible that a non-CB1-related mechanism is involved in these effects of CBD. Thus, it is worth mentioning the additional beneficial pharmacological effects of CBD, including its anti-inflammatory, antioxidant, and neuroprotective properties. These properties of CBD seem to be important not only in the context of the positive role of CBD in memory, but also in the context of cognitive disorders connected with the NMDA receptor blockade.

Such non-CB-receptor-mediated actions would explain the finding that CBD modulates a number of neurotransmitter systems, e.g., glutamatergic activity. CBD reduced glutamate-mediated neurotoxicity observed in the hippocampus and prefrontal cortex [45], as the modulation of glutamate release by cannabinoids is maintained in CB1-receptor-mutant mice [46]. Moreover, this protection of neurons from glutamate-induced death, observed after CBD administration, was unaffected by the CB receptor antagonist, indicating it to be CB-receptor-independent. Consistent with those data are our findings that a low dose of CBD (1 and 5 mg/kg) induced a tendency towards the reversal of MK-801-induced memory deficit via a possible interaction between CBD and the glutamatergic system. Thus, we may suggest that CBD may also reverse memory deficits via the glutamatergic mechanism.

Moreover, previous studies have shown that glutamate toxicity may be prevented by CBD’s antioxidant properties. As we have already mentioned, CBD was demonstrated to be an antioxidant by cyclic voltammetry, while CBD’s neuroprotective properties, mentioned below, could also be caused by an antioxidant effect. CBD, like other antioxidants, interrupts free radical chain reactions, capturing free radicals or transforming them into less active forms. An analysis of CBD’s antioxidant activity showed that it could regulate the state of redox, either directly, by affecting the components of the redox system, or indirectly, by interacting with other molecular targets associated with the redox system components. The direct antioxidant effects of CB are associated with its influence on the pro-oxidant enzyme activity, chelation of transition metal ions, interruptions of free radical chain reactions, antioxidant enzyme activity, non-enzymatic antioxidant levels, oxidative modifications of lipid protein, DNA, etc. For example, CBD reduces oxidative conditions by preventing the formation of superoxide radicals, which are mainly generated by xanthine oxidase (XO) and nicotinamide adenine dinucleotide phosphate (NADPH) oxidase (NOX1 and NOX4, respectively) [47,48]. CBD also reduces the production of reactive oxygen species (ROS) by chelating transition metal ions, involved in the Fenton reaction, to form extremely reactive hydroxyl radicals [49]. In turn, the indirect antioxidant effects of CBD are associated with its influence on AEA concentrations and, thus, on the activation/inhibition of CB receptors [50,51,52]. Interestingly, CBD was found to be more protective against glutamate toxicity than either of the dietary antioxidants—i.e., α-tocopherol or ascorbate [45]—indicating it to be a potent antioxidant. In addition, CBD strengthens the adenosine A2A receptor, which suppresses the activity of immune cells, thus protecting tissues against inflammation [53,54].

CBD has also been found to exert some agonist activity on the serotonin (5-HT) receptor type 5-HT1, in addition to inhibiting 5-HT reuptake and reducing the overall 5-HT neurotransmission [55]. However, it is not clear whether 5-HT1A’s participation in the effects of CBD is a consequence of increased serotonin availability, or of direct receptor activation/facilitation by CBD itself. Russo et al. [56] demonstrated that CBD, while showing a low-affinity agonism towards the 5-HT1A receptor, could enhance 5-HT1A-mediated neurotransmission [56].

CBD induces various pharmacological effects, possibly by enhancing 5-HT neurotransmission, followed by a subsequent activation of the postsynaptic 5-HT1A receptors. In addition to this mechanism, however, there is also some evidence that CBD can facilitate serotonin action on 5-HT1A receptors—probably through an allosteric mechanism [57]. CBD can also stimulate the 5-HT1A receptor indirectly by increasing AEA levels [58]. However, the activated 5-HT1A receptor can act as a membrane antioxidant by capturing ROS [59].

As such, the affinity of CBD for the 5-HT receptors seems to be responsible for its neuroprotective effects, but not exclusively. As we have mentioned before, CBD demonstrates anxiolytic, antidepressant, and analgesic effects, and it is known that the anxiolytic and antioxidant effects of CBD depend, at least in part, on the activation of 5-HT1A [58,60].

Our findings and the literature data cited above indicate that CBD has a supporting effect on the memory process. These beneficial effects of CBD on memory are likely dose-dependent, but the mechanism of their action is not yet entirely clear. First, it should be noted that CBD exerts a significant influence on memory and learning processes by modulating the ECS functions, where the receptors of the ECS are located, e.g., in the hippocampus, the key structure that is responsible for creating memory routes. Moreover, CBD, by acting not only on the ECS but also on the other above-mentioned receptors, can encompass more complex mechanisms of action and different targets, resulting in various behavioral effects. The influence of CBD on the dopaminergic, glutamatergic, and serotoninergic receptors, or on the inhibition of inflammatory processes in the brain and the progressive degeneration of nerve cells, may also underlie the memory improvement mechanisms.

Taking into account the broad beneficial pharmacological properties of CBD, it appears to be a potentially useful therapeutic agent for the prevention and treatment of many diseases, the etiology of which may be associated with redox imbalance, inflammation, or neurodegeneration, e.g., diabetes, diabetes-related cardiomyopathy, cardiovascular diseases (including stroke, arrhythmia, atherosclerosis, and hypertension), cancer, arthritis, anxiety, psychosis (schizophrenia), epilepsy, and neurodegenerative diseases (i.e., Alzheimer’s) [60].

## 4. Materials and Methods

### 4.1. Animals

The experiments were carried out on naive male Swiss mice (Farm of Laboratory Animals, Warszawa, Poland) weighing 20–30 g; 4 weeks of age. Mice were housed in groups of 10 mice/home cage (38 × 22 × 18 cm^3^), made of white Plexiglas. The animals were maintained under standard laboratory conditions (12 h light/dark cycle, room temperature at 21 ± 1 °C) with free access to tap water and laboratory feeding (Agropol, Motycz, Poland) in their home cages, and adapted to the laboratory conditions for at least 1 week. Each experimental group consisted of 8–10 animals. All behavioral experiments were performed between 8:00 and 15:00.

All studies were carried out according to the ARRIVE guidelines to improve the reporting of animal research and improve the quality of the studies, and were conducted in accordance with the National Institute of Health Guidelines for the Care and Use of Laboratory Animals, and with the European Community Council Directive for the Care and Use of Laboratory Animals of 22 September 2010 (2010/63/EU). Furthermore, we obtained the agreement of the Local Ethical Committee for all performed experiments: Local Ethical Committee for Animal Experiments in Lublin: Approval Code: 3/2020; Approval Date: 24 February 2020.

### 4.2. Drugs

The compounds tested were: CBD (1, 5, or 30 mg/kg) (2-[(1R,6R)-3-metylo-6-(prop-1-en-2-ylo)cykloheks-2-enylo]-5-pentylo-benzeno-1,3-diol, Tocris, Bristol, UK) and MK-801 (0.6 mg/kg) (5S-10,11-dihydro-5-metylo-5H-dibenzo[a,d]cyklohepten-5,10-imin, Tocris, USA).

CBD was suspended in a 1% solution of Tween 80 (Sigma, St. Louis, MO, USA) in saline (0.9% NaCl), and administered intraperitoneally (i.p.) at a volume of 10 mL/kg. MK-801 was dissolved in saline (0.9% NaCl) and administered (i.p.) at a volume of 10 mL/kg. Fresh drug solutions were prepared on each day of experimentation. Control groups received injections of saline with Tween 80 at the same volume and by the same route of administration. Experimental doses of CBD, as well as procedures, were selected based on the literature data [11,27,28,32]. The MK-801 dose to provoke memory impairment in PA test was selected based on our previous experiments [61,62].

### 4.3. Experimental Procedure

#### 4.3.1. Memory and Learning

In order to assess and understand the memory-related effects, we used the passive avoidance (PA) test. The PA test is commonly used to investigate emotional learning and memory processes in rodents. Depending on the used procedure, the PA test allows for the examination of different memory durations (short-term and long-term memory), as well as different memory stages (acquisition, consolidation, and retrieval). It should also be noted that, regarding the PA paradigm, response latency alterations have been thought to reflect the degree of memory; however, the emotionality (fear and/or anxiety) of animals can presumably affect their avoidance behavior [63].

In our study, we assessed fear learning and memory in the PA test. This type of memory seems to be important in the context of not only cognitive, but other emotional symptoms of schizophrenia (e.g., anxiety), as well as of side effects after using neuroleptics.

##### PA Test Apparatus

The apparatus of the PA test consisted of two-compartment acrylic boxes, each with a lit (10 × 13 × 15 cm^3^) and dark (25 × 20 × 15 cm^3^) compartment. The lit chamber was illuminated with a fluorescent light (8 W) and connected to the dark chamber, provided with an electric grid floor. The entrance of animals into the dark box was punished by an electric foot shock (0.2 mA for 2 s) [64].

##### PA Test Experimental Procedures

On the first day of training, the mice were placed individually into the lit compartment and allowed to explore it. After 30 s (a habituation period), a guillotine door was raised to allow the mice to enter the dark compartment (pre-test). When the mice entered the dark compartment, the guillotine door was closed and an electric foot shock (0.2 mA) of 2 s duration was delivered immediately to the animals via the grid floor. The latency time for entering the dark compartment was recorded (TL1). If the mouse failed to enter the dark box within 300 s, it was placed into this dark box, the door was closed, and the electric foot shock was delivered to the animal. In this case, the TL1 value was recorded as 300 s. In a subsequent trial, the same mice were again individually placed in the lit compartment of the PA apparatus. After a 30 s adaptation period in the lit (safe) chamber, the door between the compartments was raised and the time taken to re-enter the dark compartment was recorded (TL2) (test). No foot shock was applied in this trial. If the animal did not enter the dark compartment within 300 s, the test was stopped and TL2 was recorded as 300 s.

For the memory-related responses, the changes in PA performance were expressed as the differences between retention and training latencies, and defined as a latency index (LI). LI was calculated for each animal as the ratio:

LI = TL2 − TL1/TL1

TL1 = the time taken to enter the dark compartment during the pre-test;

TL2 = the time taken to re-enter the dark compartment during the test.

Depending on the used procedure, the PA test allows for the examination of different memory durations (short-term and long-term memory), according to the period between the training and the test, as well as different stages of memory (acquisition, consolidation, and retrieval), according to the drug treatment duration. When the mice were tested 24 h after TL1, their long-term fear memory was assessed. Drug administration before the first trial (pretest) should interfere with the acquisition of information; drug administration immediately before the first trial (after the pre-test) should exert an effect on the process of consolidation, while the administration of the tested compounds before the second trial (before the test) should interfere with the retrieval of memory information [65].

#### 4.3.2. Locomotor Activity

Locomotion of mice was recorded individually in round actometer cages (Multiserv, Lublin, Poland; 32 cm in diameter, two light beams) kept in a sound-attenuated experimental room. Two photocell beams, located across the axis, automatically measured the animals’ movements.

### 4.4. Treatment

#### 4.4.1. Memory-Related Responses

The first step of the experiment was designed to estimate the influence of CBD (1–30 mg/kg, i.p.) on the different stages of long-term memory in mice, using the PA test.

##### Acquisition of Memory Processes

CBD (1, 5, 30 mg/kg, i.p.) or vehicle (for the control group) were administered 30 min before the first trial (acquisition of memory), and re-tested after 24 h (long-term memory) (Table 1A).

##### Consolidation of Memory Processes

CBD (1, 5, 30 mg/kg, i.p.) or vehicle (for the control group) were administered immediately after the first trial, and re-tested after 24 h (long-term memory) (Table 1B).

##### Retrieval of Memory Processes

First, the mice were tested during the first trial. CBD (1, 5, 30 mg/kg, i.p.) or vehicle (for the control group) were injected 30 min before retrieval, and that retrieval was carried out 24 h (long-term memory) after the first trial (Table 1C).

Next, based on these pilot experiments, we chose the non-effective doses of CBD for the next experiment with MK-801.

We used a pharmacological animal model of schizophrenia, i.e., the administration of the NMDA-receptor antagonist MK-801. The used procedure is commonly accepted [15], and was confirmed in our previous experiments [17,61,62]. This experimental procedure is based on the amnestic properties of MK-801. An acute administration of MK-801 induced schizophrenia-like symptoms in mice, manifesting, among others, as cognitive disturbances (correlation with the cognitive symptoms of schizophrenia in humans).

In our previously published experiments, we confirmed that an acute injection of MK-801 at the doses of 0.3 and 0.6 mg/kg diminished both the short-term and long-term acquisition, consolidation/retention, and/or retrieval of memory and learning in the PA task [17]. Therefore, based on the results obtained from our cited experiments, an effective dose of MK-801 of 0.6 mg/kg was chosen for the provocation of cognitive symptoms typical of schizophrenia in mice.

We evaluated the influence of ineffective doses of CBD (1 and 5 mg/kg) on the memory-related disorders induced by MK-801 (0.6 mg/kg) in the PA task. The effects of all substances and ligands used before their combined administration with MK-801 were tested in a wide range of doses in the tests used. In accordance with the principles adopted in experiments of this type, in order to test their influence on the behavioral effects of another compound—i.e., MK-801 at an active dose in a given test—non-effective doses were selected in order to avoid non-specific effects resulting from the actions of a given compound. These actions, after the application of the effective dose, could overlap with the obtained effect, making it difficult to correctly interpret the obtained results.

##### Acquisition of Memory Processes

Non-effective doses of CBD (1 and 5 mg/kg) or vehicle (for the control group) were administered acutely 15 min before an acute injection of MK-801 (0.6 mg/kg) or vehicle. Fifteen minutes after the last injection, the mice were tested in the PA during first trial, and re-tested 24 h later for the assessment of long-term memory acquisition (Table 2A).

##### Consolidation of Memory Processes

Non-effective doses of CBD (1 and 5 mg/kg) or vehicle (for the control group) were administered acutely 15 min before an acute injection of MK-801 (0.6 mg/kg) or vehicle, immediately after the first trial. Then, the mice were re-tested 24 h later (Table 2B).

##### Retrieval of Memory Processes

At the beginning, the mice were tested during the first trial. Then, 24 h later, a non-effective dose of CBD (1 or 5 mg/kg, i.p.) or vehicle (for the control group) was administered 15 min before injection of MK-801 (0.6 mg/kg) or vehicle. The mice were re-tested 15 min after the last injection (Table 2C).

#### 4.4.2. Locomotor Activity

Horizontal locomotor activity was measured immediately after a single injection of CBD (1–30 mg/kg) or vehicle (for the control group). The mice were then tested immediately. Locomotor activity—i.e., the number of photocell beam breaks—was automatically recorded for 30 and 60 min.

### 4.5. Statistical Analysis

The statistical analysis was performed using one-way or two-way analysis of variance (ANOVA) for the factors of pre-treatment, treatment, and pretreatment/treatment interactions. Post-hoc comparison of means was carried out using Tukey’s test (for one-way and two-way ANOVA) for multiple comparisons, when appropriate.

The data were considered statistically significant at a confidence limit of *p* < 0.05. ANOVA with Tukey’s post-hoc test was performed using GraphPad Prism version 7 for Windows (GraphPad Software, San Diego, CA, USA, www.graphpad.com).

For the memory-related behaviors, the changes in PA performance were expressed as the difference between retention and training latencies, and were taken as an LI. LI was calculated for each animal and reported as the ratio mentioned previously [61,62,64]. For the locomotor activity, we measured the number of photocell beam breaks.

## 5. Conclusions

Our results indicate that an acute injection of CBD (30 mg/kg) improved the acquisition, consolidation, and retrieval stages of fear-related memory in mice in the PA test, while the acute injection of a non-effective dose of CBD (1 or 5 mg/kg) attenuated the memory impaired by MK-801 (0.6 mg/kg) in the consolidation and retrieval stages of memory, but not in the acquisition of fear learning and memory.

Our findings confirm that cannabinoids, such as CBD, are able to control memory and learning processes. Thus, this positive influence of CBD on either memory process formation or memory impairment by the NMDA receptor antagonist (MK 801) may offer a fairly promising perspective for research and development projects, targeting more effective pharmacotherapy of cognitive-related disorders, including the cognitive symptoms of schizophrenia.

In conclusion, several preclinical studies, including our study, demonstrate a variety of memory improvement processes after acute CBD treatment. Our results provide clear evidence for the potential role of CBD in the attenuation of cognitive decline associated with NMDA receptor dysfunction. Our findings may also have important implications for the treatment of many memory disorder conditions, such as schizophrenia. However, further studies are required in order to provide further evidence for the potential of CBD to improve cognitive, schizophrenia-related disorders in long-term protocols. Moreover, extensive randomized and controlled clinical trials would be beneficial in order to confirm that the obtained research findings are translatable to clinical practice.

## Figures and Tables

**Table 1:** The scheme of CBD or vehicle administration during the assessment of long-term memory acquisition (A), consolidation (B), or retrieval (C) in the PA test in mice; + test performed.

**A**					
**Acquisition of memory**					
**PA test**	***drug administration***	***interval***	***TL1***	***interval***	***TL2***
Long-term memory	CBD (1, 5, 30 mg/kg)	30 min	+	24 h	+
	vehicle	30 min	+	24 h	+
**B**					
**Consolidation of memory**					
**PA test**	***TL1***	***interval***	***drug administration***	***interval***	***TL2***
Long-term memory	+	0 min	CBD (1, 5, 30 mg/kg)	24 h	+
	+	0 min	vehicle	24 h	+
**C**					
**Retrieval of memory**					
**PA test**	***TL1***	***interval***	***drug administration***	***interval***	***TL2***
Long-term memory	+	24 h	CBD (1, 5, 30 mg/kg)	30 min	+
	+	24 h	vehicle	30 min	+

**Table 2:** The scheme of CBD and MK-801 co-administration during the assessment of long-term memory acquisition (A), consolidation (B), or retrieval (C) in the PA test in mice; + test performed.

**A**							
**Acquisition of Memory**							
**PA test**	***drug administration***	***interval***	***drug administration***	***interval***	***TL1***	***interval***	***TL2***
Long-term memory	CBD (1 or 5 mg/kg)	15 min	MK-801 (0.6 mg/kg) or vehicle	15 min	+	24 h	+
	vehicle (control group)	15 min	MK-801 (0.6 mg/kg) or vehicle	15 min	+	24 h	+
**B**							
**Consolidation of Memory**							
**PA test**	***TL1***	***interval***	***drug administration***	***interval***	***drug administration***	***interval***	***TL2***
Long-term memory	+	0 min	CBD (1, 5, 30 mg/kg)	15 min	MK-801 (0.6 mg/kg) or vehicle	24 h	+
	+	0 min	vehicle	15 min	MK-801 (0.6 mg/kg) or vehicle	24 h	+
**C**							
**Retrieval of Memory**							
**PA test**	***TL1***	***interval***	***drug administration***	***interval***	***drug administration***	***interval***	***TL2***
Long-term memory	+	24 h	CBD (1, 5, 30 mg/kg)	15 min	MK-801 (0.6 mg/kg) or vehicle	15 min	+
	+	24 h	vehicle	15 min	MK-801 (0.6 mg/kg) or vehicle	15 min	+

## Data Availability

Data is contained within the article.

## Sample Availability

Samples of the compounds are not available from the authors.

## Abbreviations

Δ9-THC: Δ9-tetrahydrocannabinol; 2-AG: 2-arachidonoylglycerol; 5-HT: serotonin; AEA: N-arachidonoylethanolamine; CB: cannabinoid; CBD: cannabidiol; ECS: endocannabinoid system; Glu: glutamate; LI: latency index; NADPH: nicotinamide adenine dinucleotide phosphate oxidase; NMDA: N-methyl-D-aspartate; NOR: novel object recognition; PA: passive avoidance; ROS: reactive oxygen species; TL: transfer latency; XO: xanthine oxidase; i.p.: intraperitoneally.
